# LIFE HABITS AND LIPID PEROXIDATION OF WOMEN OVER 40 YEARS OLD

**DOI:** 10.1093/geroni/igz038.533

**Published:** 2019-11-08

**Authors:** Renata F Oliveira, Maria Mota, Jorge Soares, Zirlene Santos, Bianca Rosa, Caroline Santana, Dalton Pessoa-Filho, Cassiano M Neiva

**Affiliations:** 1 Universidade de Trás-os-Montes e Alto Douro, Vila Real, Portugal, Portugal; 2 UTAD, Vila Real, Portugal, Portugal; 3 UNIFENAS, Divinópolis, Minas Gerais, Brazil; 4 UNESP, Bauru, São Paulo, Brazil

## Abstract

Oxidative stress is involved in degenerative processes, aging, and diseases. Lifestyle can be change oxidative stress. One of reactive oxygen species targets is polyunsaturated fatty acid, an important cellular membrane component. The aim of this study is to analyse the contribute of lifestyle in lipid peroxidation in over fourty years old women. Were included 60 women whith age ranged between 41 and 82 years old (53.3 ± 9.1 years). Lifestyle were explored doing a food frequency questionnaire, Perceptive Stress Scale (PSS) and International Physical Activity Questionnaire (IPAQ). The functional capacity was determinate by 6-min walking test (cardiorespiratory capacity) and Squat-jump (leg strength and power). Parameters of oxidative stress were determinate from plasma during fasting, lipid peroxidation was evaluated by TBARs, and antioxidant capacity was evaluated by catalase activity and ABTS. Spearman correlation and Multiple Linear Regression model, through Stepwise method, considering TBARs as dependent variable, and age, weight, body mass index, waist circumference, stress perception, physical activity level, total antioxidant capacity, catalase activity, cardiorespiratory capacity, leg strength and power, daily caloric intake, daily fruit, vegetables, coffee/tea, vitamin E and alcohol intake, as independent variable, was performed. Negative correlations were obtained between TBARs and cardiorespiratory capacity (r= -0.35; p=0.026) and between TBARS and ABTS (r= -0.33; p=0.038). Total antioxidant capacity was the model’s first variable (F= 5.50; p = 0.013), explaining 15.3% of TBARS, then cardiorespiratory capacity (F= 5.50; p = 0.047), explaining 10.5% of TBARs The results revealed total antioxidant capacity and cardiorespiratory capacity as predictors to lipid peroxidation damage.

